# A Multicenter Study of Factors Related to Early Implant Failures—Part 2: Patient Factors

**DOI:** 10.1111/cid.70081

**Published:** 2025-07-29

**Authors:** Rachel Duhan Wåhlberg, Victoria Franke Stenport, Ann Wennerberg, Lars Hjalmarsson

**Affiliations:** ^1^ Department of Prosthodontics/Dental Materials Science Institute of Odontology, The Sahlgrenska Academy, University of Gothenburg Gothenburg SE Sweden; ^2^ Specialist Dental Clinic, Folktandvården Sörmland AB, The Mälar Hospital Eskilstuna Sweden; ^3^ Center for Clinical Research Sörmland, Uppsala University Eskilstuna Sweden

**Keywords:** early implant complications, early implant failure, multicenter cohort study, multivariable regression analysis

## Abstract

**Background:**

Recent advancements in dental implant materials, designs, and surgery have increased their use, especially for challenging local conditions. As guidelines for individualized risk assessment are increasingly emphasized, implant treatment has become available to patients with diverse medical backgrounds. However, clinical research with large patient groups is needed to investigate the effects of patient‐related factors associated with early implant failure.

**Purpose:**

This paper investigates patient factors in two patient cohorts associated with early implant complications and failures.

**Materials and Methods:**

The collected data were analyzed and presented in two studies. Both studies followed the same data collection methodology and compared cohorts treated in 2007 and 2017. The same patient‐level dataset was analyzed, although the second study included additional analyses of diseases and allergies. Data were analyzed univariately (*p* < 0.20) to select variables for the multivariable logistic regression model (*p* < 0.05), with early implant failures and complications as dependent variables.

**Results:**

In total, 1875 patients with 4670 implants were included. There were 74 (3.7%) dropouts, mainly due to lack of data. The 2007 cohort comprised 799 patients with 2473 implants, and the 2017 cohort comprised 1076 patients with 2287 implants. Differences were observed between the two cohorts for the number of implants per patient, exposed implant threads, and preoperative antibiotics. In the 2007 cohort, 23 (2.9%) patients had early implant failure. In the 2017 cohort, 40 (3.7%) had early implant failure (*p* > 0.30). Significantly more implants failed in the 2017 cohort (*n* = 56, 2.4%) than in the 2007 cohort (*n* = 26, 1.1%) (*p* < 0.001). Early complications were reported for 56 (7.0%) patients in 2007 and 145 (13.5%) patients in 2017 (*p* < 0.001). Three patient‐related variables were associated with an increased risk of early failure—food allergy, exposed implant threads, and increased number of implants. Seven variables were related to an increased risk of complications: smoking, exposed threads, no preoperative antibiotics, number of implants, sinus perforations, food allergy, and metal allergy.

**Conclusions:**

This study identified three factors associated with early implant failure and seven associated with early complications.

## 1 Introduction

Dental implant treatment, a widely used and effective method for replacing missing teeth [1], was initially developed for the rehabilitation of edentulous jaws using full‐arch prosthetic restorations [2, 3]. Since then, progress has been made in the dental implant field. Today, dental implant surgery includes implant treatment in partial edentulous patients, which is now the largest patient group [4]. Over time, clinical guidelines have placed greater emphasis on individualized evaluation of the patient's health condition to create a more holistic view of the patient's needs and assess the potential benefit of implant therapy [5, 6, 7, 8]. Research and development of surgical methods, materials, and designs have enabled local management of sites with poor bone quality and insufficient bone volume [9]. Consequently, the indications for implant treatment have expanded lately. As implant treatment is offered to patients with varied medical backgrounds, patient‐related risk factors need to be addressed. Future research should focus on long‐term predictable results and prevention of early implant failure and complications [9, 10].

Dental implants, which can immediately activate a foreign body reaction [11], trigger the immunological system to suppress bone resorption and stimulate bone formation to isolate the implant [11, 12], resulting in osseointegration. After osseointegration, the implant is covered in bone; however, a few implants are isolated by fibrotic encapsulation [12]. Most implant failures occur during the early healing phase [13, 14, 15], which is referred to as early implant failures. Early implant failures as the result of unsuccessful osseointegration are often related to patient‐related factors that cause an immunological imbalance [10, 16], resulting in an over activation of the immune system [17, 18]. This effect on the immune system highlights why further investigations that examine systemic health conditions as potential risk factors for implant failure are needed. Although some medical conditions make implant treatment too risky [19], most systemic conditions are seen as having low relative risks [5, 6]. Conditions such as cardiovascular diseases, osteoporosis, diabetes, and autoimmune diseases warrant careful assessment but do not automatically exclude patients from implant treatment [5, 20, 21, 22]. When potential medical complications are present, a cautious and a well‐informed decision‐making process is recommended [8], favoring individualized assessment over the application of absolute contraindications. In a large retrospective case–control study, Olsson Malm et al. identified several anamnestic factors, including systemic diseases, allergies in general, and food allergies specifically, that increased the probability of early implant failure [10]. The influence of patients' general health on early implant failure is poorly understood and therefore needs further study [20, 23, 24].

Early implant failure has frequently been associated with impaired bone healing and reduced primary stability [1, 23]. Previous studies have shown that smoking [10, 25, 26, 27, 28] and implant sites with poor bone quality or small bone volume [13, 29] are risk factors for early failure. Other local conditions involve implant placement. Most studies show that implants are more prone to fail in the maxillae due to poorer bone quality, challenging sinus anatomy, and often small vertical bone height [13, 23]. Although early implant failure in the maxilla versus mandible has been widely studied, findings remain inconsistent when jaws are divided into different regions [23].

Although risk factors for early implant failure have been studied, few studies include large patient samples with two cohorts. Systematic immune responses related to a patient's general health and local complications associated with the risk of early implant failure are understudied areas. This study aims to evaluate patient‐related factors and early implant failure and complications in one patient cohort treated in 2007 and one patient cohort treated in 2017.

## 2 Materials and Methods

This retrospective cohort study is based on patient‐related information on performed implant surgeries at three specialist centers in the region of Västra Götaland in Sweden between 2007 and 2017. Due to the extensive dataset, this multicenter study was structured into two parts. Part I focuses on implant materials, design, and surgical technique [30] and Part II, the present study, focuses on patient‐related factors.

### 2.1 Data

Part I and Part II were conducted using the same underlying dataset. The methodology for data collection was described in Part I [30]. In Part II, data on specific diseases and allergies was analyzed to provide more detailed information on general health conditions, including cardiovascular disease, diabetes, autoimmune disorders, and allergies to food, antibiotics, and metals. The selected conditions were commonly reported and considered clinically relevant, as they may have systemic effects on the foreign body response to implants [6, 25, 31, 32] Furthermore, descriptive analyses of implant placement by jaw region were conducted in cases of implant failure and complications, as well as analyses of the number of implants placed per surgical procedure.

### 2.2 Definition

This study defines early implant failures and complications as failures and complications within the first year.

### 2.3 Statistics

Statistical data analyses were performed in SPSS (IBM SPSS Statistics for Windows, Version 28.0. Armonk, NY: IBM Corp). Descriptive statistics were used for numbers, means, and frequencies. Fisher's exact test and Fisher's permutation test were used to compare the outcome of binary and continuous variables of the two cohorts (*p* < 0.05). Patients with implants placed in the maxilla were compared with those with implants in the mandible, and similarly, patients with implants in one region were compared with those with implants in other regions, with respect to implant failure and complications. For patients who received implants in both the maxilla and mandible, or across multiple regions, the proportion of implants placed in the maxilla or in each specific region was calculated. All statistical analyses were conducted at the patient level; implant‐level data were used solely for descriptive purposes, as shown in Table 2. Furthermore, the number of surgical procedures was described in three categories based on the Swedish coding system: 1, 2 to 3, and 4 or more implants inserted simultaneously per patient per day.

In the univariate analysis in Part II, the same patient‐level variables described in Part I were used as the basis for variable selection in the subsequent multivariable logistic regression analysis. Additionally, variables of cardiovascular disease, diabetes, autoimmune disorders, and allergies to food, antibiotics, and metals were included. Early implant failures and complications were classified as dependent variables; all others were classified as independent variables. In the univariate analysis, all variables with a significance level of *p* < 0.2 were compared for further variable selection and inclusion in the multivariable models. Factors associated with an increased risk of early implant failure and complications were analyzed with a significance level of *p* < 0.05 in a multivariable logistic regression model.

### 2.4 Ethical Protection

The study was approved by the Ethical Review Board, Sweden (2019–01330).

## 3 Results

### 3.1 Patients Inclusion and Exclusion

Of the 1949 patients treated with implants, 862 patients were from the 2007 cohort and 1087 were from the 2017 cohort. Of these, 74 (3.8%) patients were excluded from the study–63 patients from 2007 and 11 patients from 2017–for the following reasons: surgical reports not found (52 patients from 2007 and 8 patients from 2017); and incorrect year entered for treatment (11 patients from 2007 and 3 patients from 2017). Moreover, zygoma implants were excluded due to deviant design and surgical technique (2 implants from 2007). In total, 1875 patients with 4670 implants were included–799 patients with 2473 implants from 2007 and 1076 patients with 2287 implants from 2017.

### 3.2 Patient‐Related Factors for 2007 and 2017 Cohorts

Information on reported patient characteristics and medical history, bone status, implant jaw placement, and other patient‐related variables were compared for the two cohorts (Table 1). Subside analyses were conducted for circulatory diseases, which included hypertension, dyslipidemia, coronary infarction, stroke, angina pectoris, transient ischemic attack (TIA), pulmonary embolism, valve insufficiency, and heart arrhythmia. Subside analysis of autoimmune diseases includes Sjögren's syndrome, rheumatoid arthritis, systemic lupus erythematosus (SLE), spondylitis, Bechterew's disease, psoriasis, ulcerative colitis, Crohn's disease, polymyalgia, multiple sclerosis, thyroid diseases, celiac disease, and diabetes type 1.

**TABLE 1:** Summary of variables, analyzed at the patient level for each cohort, 2007 and 2017.

Variable			2007 versus 2017
	2007	2017	Two‐tailed *p*
Gender (women), *n* (%)	443 (55.4)	596 (55.4)	> 0.30
Age Mean ± SD (min; max)	55.7 ± 22.1 (16; 96)	54.0 ± 18.2 (17; 98)	0.068
Age groups, *n* (%)			
< 20	126 (15.8)	36 (3.3)	
20–39	86 (10.8)	185 (17.2)	
40–59	115 (14.4)	372 (34.6)	
60–79	401 (50.2)	431 (40.1)	
> 80	71 (8.9)	52 (4.8)	
Smoking, *n* (%)	123 (15.4)	157 (14.6)	> 0.30
Diseases, *n* (%)	416 (52.1)	580 (53.9)	> 0.30
Subgroup: Circulatory diseases	228 (28.5)	255 (23.7)	**0.019**
Subgroup: Diabetes type 1 and 2	47 (5.9)	64 (5.9)	> 0.30
Subgroup: Autoimmune diseases	56 (7.0)	82 (7.6)	> 0.30
Allergies, *n* (%)	221 (27.7)	300 (27.9)	> 0.30
Subgroup: Antibiotic allergy	81 (10.1)	110 (10.2)	> 0.30
Subgroup: Food allergy	37 (4.6)	58 (5.4)	> 0.30
Subgroup: Metal allergy	17 (2.1)	22 (2.0)	> 0.30
Bone quality mean ± SD (min; max)^a^	2.72 ± 0.59 (1; 4)	2.67 ± 0.66 (1; 4)	0.12
Missing data, *n* (%)	167 (20.9)	536 (49.8)	
Bone volume mean ± SD (min; max)^b^	2.33 ± 0.62 (1; 4)	2.34 ± 0.59 (1; 5)	> 0.30
Missing data, *n* (%)	165 (20.7)	621 (57.7)	
Primary stability mean ± SD (min; max)^c^	1.0 ± 0.1 (1; 3)	1.0 ± 0.1 (1; 3)	> 0.30
Missing data, *n* (%)	404 (50.6)	578 (53.7)	
Number of implants per patient mean ± SD	3.1 ± 2.1	2.1 ± 1.7	**< 0.001**
Exposed implant threads, *n* (%)	13 (1.6)	60 (5.6)	**< 0.001**
Bone augmentation, *n* (%)	92 (11.5)	152 (14.1)	0.11
Sinus perforations, *n* (%)	6 (0.8)	7 (0.7)	< 0.30
Implant jaw placement (maxillae), *n* (%)	495 (62.0)	675 (62.7)	> 0.30
Preoperative antibiotics	742 (92.9)	835 (77.6)	**< 0.001**
Early implant failure, *n* (%)	23 (2.9)	40 (3.7)	> 0.30
Early implant complications, *n* (%)	56 (7.0)	145 (13.5)	**< 0.001**

*Note:* Results from Fisher's exact test and Fisher's permutation test reported as two‐tailed *p*‐values. Level of significance *p* < 0.05. Bold font indicates statistically significant values.

^a^ Bone quality is reported on a 1–4 ordinal scale, analyzed stepwise for each 1 (one) unit difference.

^b^ Bone volume is reported on a 1–5 ordinal scale, analyzed stepwise for each 1 (one) unit difference.

^c^ Primary stability is reported on a 1–3 ordinal scale, analyzed stepwise for each 1 (one) unit difference.

In 2017, implant insertions in patients aged 20 to 59 years increased compared with 2007, while treatment in the youngest patients, under 20 years old, and the oldest patients, 60 years or older, decreased. Bone quality was reported as I–IV, ranging from homogenous cortical bone to thin cortical bone with low‐density trabeculae, and bone volume was reported as A–E, ranging from minimal to extreme jaw resorption per Lekholm and Zarb's classification system [33]. Primary stability was defined as good, moderate, or poor. These variables were not completely reported by all clinics. No statistical difference was observed for bone quality, bone volume, or primary stability between the cohorts (*p* < 0.30). More patients had one or more implants with exposed implant threads in 2017 (*n* = 60, 5.6%) than in 2007 (*n* = 13, 1.6%) (*p* < 0.001). Fewer patients received prescribed antibiotics before implant treatment in 2017 (*n* = 835, 77.6%) than in 2007 (*n* = 742, 92.9%) (*p* < 0.001) (Table 1).

Implant surgeries were recorded as 1, 2 to 3, or 4 or more implants per patient per day. The number of performed single implant surgeries was higher in 2017 (*n* = 853) than in 2007 (*n* = 390). Likewise, the number of surgeries for two to three implants placed simultaneously was higher in 2017 (*n* = 340) than in 2007 (*n* = 308), but fewer four or more implant surgeries were performed in 2017 (*n* = 146) than in 2007 (*n* = 276).

Most implants were placed in the maxilla for both the 2007 cohort (*n* = 1534, 62.0%) and the 2017 cohort (*n* = 1407, 61.5%) (*p* < 0.30). More implants were placed in the molar region in 2017 (*n* = 546, 23.9%) than in 2007 (*n* = 195, 7.9%) (*p* > 0.001). Conversely, the number of implants placed in the incisor and canine region was lower in 2017 (*n* = 739, 32.3%) than in 2007 (*n* = 1107, 44.8%) (*p* > 0.001). Similarly, fewer implants were placed in the premolar region in 2017 (*n* = 1002, 43.8%) than in 2007 (*n* = 1171, 47.4%) (*p* = 0.0067).

### 3.3 First Year Implant Loss and Complications

In 2017, 40 (3.7%) patients had implant failure compared with 23 (2.9%) in 2007 (*p* < 0.3). More implants failed in 2017 (*n* = 56, 2.4%) than in 2007 (*n* = 26, 1.1%) (*p* < 0.001). Complications were more frequently reported at both the patient and implant level in 2017 than in 2007. In 2017, 145 (13.5%) patients with 241 (10.5%) implants had complications; in 2007, 56 (7.0%) patients with 94 (3.8%) implants (*p* < 0.001) had complications. The cause of early failures and the reported complications are described in the first part of the study [30].

The following data combines the cohorts. Patients with implant failures had a mean number of 3.7 implants per patient, and patients with no implant failure had a mean number of 2.5 implants per patient (*p* < 0.001). Implant failures of one implant per patient were more prevalent (*n* = 51) than multiple implant failures (*n* = 12). For patients who lost all their implants, 12 patients lost 1 of 1 implant, three patients lost 2 of 2 implants, and one patient lost 6 of 6 implants.

Univariate, patient‐level analyses of early failures and complications related to implant placement revealed no statistically significant differences between patients with implants placed in the maxilla versus the mandible (*p* ≥ 0.25), or between those with implants placed in a specific region compared with all other regions. Patients with implants in the anterior region reported a higher frequency of early complications (*p* = 0.086), while failures and complications in other regions were further from statistical significance (*p* ≥ 0.28).

In a multivariable logistic regression model, neither jaw placement (maxilla vs. mandible) nor region placement (incisor, premolar, molar) was significantly associated with an increased risk of early implant failure or complications (*p* ≥ 0.25). Descriptively, the distribution of implant placement was presented at the implant level (Table 2).

**TABLE 2:** Description of implant placement in cases with reported early complications and failures, described at the implant level in both cohorts.

Implant placement	Implants *n*	Failure *n* (%)	Failure %	Complications *n* (%)	Complication %
Jaw placement					
Maxillae	2941	59 (72.0)	2.0	217 (64.8)	7.4
Mandible	1819	23 (28.0)	1.3	118 (35.2)	6.5
Jaw region placement					
Incisor region	1846	38 (46.3)	2.1	144 (43.0)	7.8
Maxillary incisor	1410	30 (36.6)	2.1	111 (33.1)	7.9
Mandible incisor	436	8 (9.8)	1.8	33 (9.9)	7.6
Premolar region	2173	34 (41.5)	1.6	144 (43.0)	6.6
Maxillary premolar	1236	25 (30.5)	2.0	85 (25.4)	6.9
Mandible premolar	937	9 (11.0)	1.0	59 (17.6)	6.3
Molar region	741	10 (12.2)	1.3	47 (14.0)	6.3
Maxillary molar	295	4 (4.9)	1.4	21 (6.3)	7.1
Mandible molar	446	6 (7.3)	1.3	26 (7.8)	5.8

### 3.4 Factors Associated With Early Failure and Complications

The multivariable logistic regression analysis of early failures identified three variables associated with increased risk of early failure: bone perforations and exposed implant threads (OR 3.47; 95% CI [1.56, 7.71]; *p* < 0.0023), increased number of implants (OR 1.27; 95% CI [1.15, 1.41]; *p* < 0.001) and food allergy (OR 2.64; 95% CI [1.15, 6.06]; *p* = 0.022) (Table 3). Regarding early complications, seven variables were found to be associated with an increased risk in the multivariable analysis: smoking (OR 2.30; 95% CI [1.61, 3.29]; *p* < 0.001), no pre‐operative antibiotics (OR 1.92; 95% CI [1.33, 2.78]; *p* < 0.001), increased number of implants (OR 1.21 95% CI [1.13, 1.30]; *p* < 0.001), exposed implant threads (OR 4.46; 95% CI [2.59, 7.69]; *p* < 0.001), sinus perforations (OR 8.19; 95% CI [2.45, 27.35]; *p* < 0.001), food allergy (OR 1.92; 95% CI [1.08, 3.40]; *p* = 0.026) and metal allergy (OR 2.45; 95% CI [1.08, 5.57]; *p* = 0.033) (Table 3).

**TABLE 3:** Multivariable logistic regression analyses on early implant failure and complications, analyzed at the patient level in both cohorts.

	Odds ratio (95% CI)	Two‐tailed *p*
Early Failures		
Diseases	1.46 (0.84, 2.52)	0.18
Smoking	1.61 (0.88, 2.94)	0.12
Bone quality^a^	1.50 (0.91, 2.47)	0.12
Numbers of implants per patient^b^	**1.27 (1.15, 1.41)**	**< 0.001**
Bone augmentation	1.25 (0.63, 2.48)	> 0.30
Exposed threads	**3.47 (1.56, 7.71)**	**0.0023**
Sinus perforations	3.41 (0.68, 16.98)	0.13
Diabetes	1.84 (0.81, 4.19)	0.15
Food allergy	**2.64 (1.15, 6.06)**	**0.022**
Antibiotic allergy	1.33 (0.64, 2.77)	0.45
Cardiovascular diseases	1.31 (0.76, 2.25)	0.34
Early complications		
Diseases	1.25 (0.91, 1.72)	0.17
Smoking	**2.30 (1.61, 3.29)**	**< 0.001**
No preoperative antibiotics	**1.92 (1.33, 2.78)**	**< 0.001**
Number of implants per patient^b^	**1.21 (1.13, 1.30)**	**< 0.001**
Bone augmentation	1.00 (0.64, 1.57)	> 0.30
Exposed threads	**4.46 (2.59, 7.69)**	**< 0.001**
Sinus perforations	**8.19 (2.45, 27.35)**	**< 0.001**
Circulatory diseases	1.36 (0.97, 1.90)	0.073
Food allergy	**1.92 (1.08, 3.40)**	**0.026**
Metal allergy	**2.45 (1.08, 5.57)**	**0.033**

*Note:* Level of significance *p* < 0.05. Bold font indicates statistically significant values.

^a^ Bone quality is reported on a 1–4 ordinal scale, analyzed stepwise for each 1 (one) unit difference.

^b^ Number of implants per patient analyzed a for each 1 (one) implant unit difference.

## 4 Discussion

This multicenter study investigates factors associated with early implant failure and complications in two patient cohorts. Part I of this study investigates implant material and design and surgical techniques [30], and Part II of this study identifies patient factors. In Part II, exposure of implant threads, food allergies, and increased numbers of implants per patient were identified as risk factors for early implant failure. Smoking, no use of preoperative antibiotics, increased number of implants, exposed implant threads, sinus perforations, food allergy, and metal allergy were identified as risk factors for early complications as they may influence the healing process and the immunological response. Growing evidence emphasizes the role of the immunological system in the osseointegration process of dental implants [11, 16, 17, 34]. In an in vivo study, Trindade et al. demonstrated that titanium implants activate the immune system [11]. Interestingly, we found that food allergy is associated with an increased risk for early implant failure. Olsson Malm et al., in a large retrospective case–control study, noted that the relation between implant failure and food allergy has not been widely discussed in the literature [10]. However, previous studies have established that food antigens upregulate immunological markers that maintain allergic inflammation [35, 36, 37, 38]. The key cytokines in food allergy—interleukin‐4 (IL‐4), IL‐5, and IL‐13—operate in peripheral tissues upon inflammatory stimuli [36, 39, 40]. Excessive cytokine production in wound repair may delay the process and possibly mediate a fibrotic wound healing [41, 42]. More research is needed to establish the clinical correlation between food allergy and early implant failure; however, upregulated immunological activation can exacerbate the inflammatory response to dental implants and mediate fibrotic encapsulation and affect osseointegration.

The subside analysis showed a correlation between metal allergy (i.e., the implants) and increased risk for early complications, a finding that could be the result of proinflammatory cytokines, an innate immune system response, reacting to the presence of the implants [43]. That is, excessive production of proinflammatory cytokines can increase the inflammatory response to the implants. The proinflammatory cytokines IL‐1β, TNF‐α, and IL‐6 have been used as biomarkers to investigate periimplant diseases [44] and are also produced in pathological allergic contact dermatitis [45]. High levels of these proinflammatory cytokines have been correlated with patient‐reported pain during inflammation [43, 46]. In addition, patients who are sensitive to one metal may also be sensitive to other metals [47]. It is possible that individuals reporting any kind of metal allergy are more attentive to postoperative symptoms following titanium implant placement. Although the evidence for titanium allergy is weak [48], trace amounts of other metals present in titanium implants may cause hypersensitivity reactions [49, 50]. A previous review article concluded that caution should be exercised when considering implant treatment for patients with any history of reported allergies [32].

More patients received implants but fewer implants were inserted per patient in 2017 (2.1) than in 2007 (3.1) (*p* < 0.001). Fewer implants were placed in the anterior (*p* < 0.001) and premolar region (*p* = 0.0067), but more implants were placed in the molar region (*p* < 0.001) in 2017 than in 2007. In Sweden, the regulations for government subsidies for implant placement in the molar region were changed 2014 [51]. Reduced patient cost for tooth replacement in the molar region may explain higher insertions in that region in 2017. Lower social acceptance for tooth gaps and extended patient inclusion criteria may have increased the demand for restorative treatment in single or minor partial tooth gaps in 2017. Fewer implants were inserted simultaneously in 2017, with fewer surgeries of four or more implants than in 2007. The placement of four or more implants per jaw is commonly used for full jaw replacement, a procedure that has become rare in Sweden as more people have their own teeth later in life [52].

There were no statistically significant differences between the cohorts regarding the number of patients who experienced early implant failure (*p* > 0.3) but more implants were lost in 2017 (*p* < 0.001) than in 2007. Statistically significantly more major and minor postoperative complications were reported in 2017 (*p* < 0.001) than in 2007. However, there may be an underestimation of reported complications in 2007 as the documentation was less detailed in 2007 than in 2017.

The fundamental background variables, including gender distribution, smoking habits, and the incidence of general diseases and allergies, were comparable between cohorts, with no statistically significant differences observed. Cardiovascular diseases were reported more frequently in 2007. Overall, the similarity in these patient characteristics supports cohort comparability and suggests that other factors account for the increased rate of failures and complications reported in 2017. One notable difference between the cohorts was the incidence of exposed implant threads: more threads were exposed in the 2017 cohort (*n* = 60, 5.6%) than in the 2007 cohort (*n* = 13, 1.6%) (*p* < 0.001). Bone perforations and exposed implant threads significantly increased the risk of early failure and complications. Previous studies found high survival rates for implants with minor dehiscence; however, exposed threads have been shown to increase the risk of vertical bone loss at the buccal aspect of the implant [53, 54, 55]. Higher incidence of exposed threads may indicate implant insertions in implant sites with reduced bone volume. Resorbed ridges and reduced bone height in the posterior maxillae increase the risk of hard tissue defects when implants are not fully embedded in bone [56]. However, at the patient level, the analyses revealed no statistically significant differences in bone volume between the cohorts. Nonetheless, the results for the bone status variables–that is, bone volume, bone quality, and primary stability–must be interpreted carefully as these variables were not fully reported by all clinics. Moreover, single implant surgeries were more frequent in 2017, but the number of full jaw surgeries decreased compared with 2007. Implant surgery in single tooth gaps may pose a greater challenge than surgery for full jaw replacement as the direction and position of own teeth must be considered for successful prosthetic rehabilitation. Implants placed in an advantageous position for prosthetic treatment are more likely to get bone dehiscence [56]. Moreover, implant placement in edentulous jaws may be preceded by reduction of the crestal bone and thin bone irregularities for decreased risk of small bone defects.

Patients with implant failure had a mean number of one more implant per patient compared with patients with no implant failure (*p* < 0.001). The multivariable logistic analysis showed that increased numbers of implants per patient had a statistically significant increase in the risk for failure (27% per implant). This finding is aligned with findings in other studies. In a large retrospective study of partially edentulous patients, Jemt et al. showed that the risk of implant failure increases by 25% for each additional implant placed during surgery in the first year [57]. It has been suggested that patient‐related factors influence the risk of tooth and implant loss in addition to the hazard ratio, which increases with the number of implants inserted [58, 59, 60].

Most studies have reported higher implant failure rates in the maxilla than in the mandible [13, 14]. Similarly, this study observed a comparable trend, with more failed implants in the maxilla. However, the difference in failure rates at the patient level between those with implants in the maxilla and those with implants in the mandible did not reach statistical significance. Furthermore, no statistically significant differences were found in implant failure or complications related to implants placed in the incisor, premolar, or molar regions. Different implant sites present distinct challenges at implant insertion related to individual bone biology and anatomy. The posterior maxillae have the lowest bone density, and implant failures are often observed in that region due to worse primary stability and greater chewing forces [61]. Moreover, the anatomical structures of the maxillary sinus may complicate the placement of implants in the premolar or molar region. However, findings in previously published studies are diverse. The mandibular anterior region, with its cortical bone and less blood supply, has also been associated with the highest rates of early implant failures [58]. Moreover, a thin buccal plate with a risk for bone resorption or soft tissue recession may be evident for implant placement in the maxillary anterior region [62, 63]. Although not statistically significant, this study observed a potential trend toward more complications in the incisor regions of the maxilla and mandible (*p* = 0.086). Kahn et al. reported that implant placement in this area may lead to postoperative eating difficulties [64]. This is also an aesthetically sensitive region [62, 63], as it is visible to the patient. Patients may become more attentive and concerned about postoperative symptoms and defects in this region. This increased sensitivity likely increases the chance of return visits and noted postoperative complications.

A notable decrease in the number of implant insertions was observed in the youngest age group of patients (i.e., younger than 20 years old) in 2017. This decrease may reflect the effect of changed regulations by the Swedish Social Insurance Agency. Dental care was free to patients up to 19 years of age in 2007, but the age limit was extended to 24 years in 2017 [65]. As implants inserted in growing jaws may require positional modifications [66], it may be justified to delay that treatment. Implant treatment in young patients may have been performed earlier in 2007 due to cost regulations, while delayed in 2017 for biological reasons.

Other identified risk variables that may affect the healing process and prolong the recovery time are smoking, sinus membrane perforations, and no preoperative antibiotics. Smoking has been considered a risk factor in many other studies [25, 26, 27, 28]. Cigarette smoking affects both the innate and adaptive immune system, leading to proinflammatory responses and dysfunction of immune cells [67]. Sinus membrane perforation has been associated with postoperative infection and chronic sinusitis as it may induce a bacterial pathway and compromise the stability of graft material [68, 69]. Our study shows a high odds ratio for sinus membrane perforations associated with an increased risk of complications. However, few observations and large confidence intervals mean that these findings should be viewed cautiously. Preoperative antibiotics may help control the inflammatory response and prevent infections by reducing the bacterial load [70]. According to the findings in this study, it can be assumed that compromised patients may benefit from antibiotic prophylaxis; however, the use of preoperative antibiotics is controversial. As antibiotic resistance is a concern, careful use of antibiotics should be maintained [7, 70, 71].

This study suggests that there is no relationship between cardiovascular diseases, autoimmune diseases, or diabetes and an increased risk for early implant failure or complications. As long as necessary precautions are observed, these diagnoses may not be contraindications to implant treatment. This finding is aligned with other studies, suggesting that medically well‐controlled patients do not have an increased risk for early implant failure [20, 22, 72]. However, the extent of underlying medical history is difficult to fully capture in a retrospective study like this, which relies on patient self‐reporting and surgeon documentation. Complex variables may be oversimplified when categorized or dichotomized into binary outcomes, and disease severity is not accounted for. Nonetheless, individual assessment before implant treatment based on medical history should be emphasized [19]. As more patients become implant candidates due to increased demand and advancements in the field, the challenge will be to understand how patient‐related risk factors may affect early implant failure and complications. Although early implant failure is uncommon, an overly generous approach to implant therapy may result in higher numbers of early implant failures and complications. Further research is needed to investigate the suggested connection between the observed factors and an over activated immune response as a potential cause of early implant failure.

## 5 Conclusions

- The results indicate that patients with food allergy, exposed implant threads, and an increased number of implants tend to have a greater risk of early implant failure and complications.
- An association has been observed for seven variables and an increased risk of early complications. Less use of preoperative antibiotics and more incidences of implant thread exposure seem to have contributed to the increased number of patients in the 2017 cohort who reported early complications.
- The identified variables underline the importance of being aware of local and systemic conditions and maintaining stringent patient inclusion criteria for implant treatment.

## Author Contributions

**Rachel Duhan Wåhlberg:** concept and design, data collection, planning of statistics, data analysis in collaboration with biostatistician, data interpretation, drafting of article, critical revision, and approval of article. **Victoria Franke Stenport:** concept and design, funding secured by scholarship from government research support in Public Dental Service Region Västra Götaland (TUA), planning of statistics, data interpretation, critical revision, and approval of article. **Ann Wennerberg:** concept and design, funding secured by scholarship from The Swedish Research Council, and critical revision. **Lars Hjalmarsson:** concept and design, planning of statistics, data interpretation, critical revision, and approval of article.

## Conflicts of Interest

The authors declare no conflicts of interest.

## Data Availability

The data that support the findings of this study are available from the corresponding author upon reasonable request.

## References

[1] R. I. Grigoras , A. Cosarca , and A. Ormenișan , “Early Implant Failure: A Meta‐Analysis of 7 Years of Experience,” Journal of Clinical Medicine 13, no. 7 (2024): 1887. 38610652 10.3390/jcm13071887 PMC11012615

[2] P. I. Brånemark , B. O. Hansson , R. Adell , et al., “Osseointegrated Implants in the Treatment of the Edentulous Jaw. Experience From a 10‐Year Period,” Scandinavian Journal of Plastic and Reconstructive Surgery 16 (1977): 1–132. 356184

[3] R. Adell , B. Eriksson , U. Lekholm , P. I. Branemark , and T. Jemt , “Long‐Term Follow‐Up Study of Osseointegrated Implants in the Treatment of Totally Edentulous Jaws,” International Journal of Oral & Maxillofacial Implants 5, no. 4 (1990): 347–359. 2094653

[4] R. W. Chan and T. N. Tseng , “Single Tooth Replacement‐Expanded Treatment Options,” Australian Dental Journal 39, no. 3 (1994): 137–149. 8067929 10.1111/j.1834-7819.1994.tb03082.x

[5] D. Hwang and H. L. Wang , “Medical Contraindications to Implant Therapy: Part II: Relative Contraindications,” Implant Dentistry 16, no. 1 (2007): 13–23. 17356368 10.1097/ID.0b013e31803276c8

[6] D. van Steenberghe , M. Quirynen , L. Molly , and R. Jacobs , “Impact of Systemic Diseases and Medication on Osseointegration,” Periodontology 2000 33 (2003): 163–171. 12950849 10.1046/j.0906-6713.2003.03313.x

[7] B. Klinge , T. Flemming , J. Cosyn , et al., “The Patient Undergoing Implant Therapy. Summary and Consensus Statements. The 4th EAO Consensus Conference 2015,” Clinical Oral Implants Research 26, no. Suppl 11 (2015): 64–67. 26385621 10.1111/clr.12675

[8] D. L. Cochran , S. Schou , L. J. Heitz‐Mayfield , M. M. Bornstein , G. E. Salvi , and W. C. Martin , “Consensus Statements and Recommended Clinical Procedures Regarding Risk Factors in Implant Therapy,” International Journal of Oral & Maxillofacial Implants 24, no. Suppl (2009): 86–89. 19885436

[9] D. Buser , L. Sennerby , and H. De Bruyn , “Modern Implant Dentistry Based on Osseointegration: 50 Years of Progress, Current Trends and Open Questions,” Periodontology 2000 73, no. 1 (2017): 7–21. 28000280 10.1111/prd.12185

[10] M. O. Malm , T. Jemt , and V. F. Stenport , “Patient Factors Related to Early Implant Failures in the Edentulous Jaw: A Large Retrospective Case‐Control Study,” Clinical Implant Dentistry and Related Research 23, no. 3 (2021): 466–476. 33999522 10.1111/cid.13009

[11] R. Trindade , T. Albrektsson , S. Galli , Z. Prgomet , P. Tengvall , and A. Wennerberg , “Osseointegration and Foreign Body Reaction: Titanium Implants Activate the Immune System and Suppress Bone Resorption During the First 4 Weeks After Implantation,” Clinical Implant Dentistry and Related Research 20, no. 1 (2018): 82–91. 29283206 10.1111/cid.12578

[12] T. Albrektsson , C. Dahlin , T. Jemt , L. Sennerby , A. Turri , and A. Wennerberg , “Is Marginal Bone Loss Around Oral Implants the Result of a Provoked Foreign Body Reaction?,” Clinical Implant Dentistry and Related Research 16, no. 2 (2014): 155–165. 24004092 10.1111/cid.12142

[13] B. Friberg , T. Jemt , and U. Lekholm , “Early Failures in 4,641 Consecutively Placed Brånemark Dental Implants: A Study From Stage 1 Surgery to the Connection of Completed Prostheses,” International Journal of Oral & Maxillofacial Implants 6, no. 2 (1991): 142–146. 1809668

[14] T. Jemt , “Implant Survival in the Edentulous Jaw‐30 Years of Experience. Part I: A Retro‐Prospective Multivariate Regression Analysis of Overall Implant Failure in 4,585 Consecutively Treated Arches,” International Journal of Prosthodontics 31, no. 5 (2018): 425–435. 30180226 10.11607/ijp.5875

[15] T. Jemt , “Implant Survival in the Edentulous Jaw: 30 Years of Experience. Part II: A Retro‐Prospective Multivariate Regression Analysis Related to Treated Arch and Implant Surface Roughness,” International Journal of Prosthodontics 31, no. 6 (2018): 531–539. 30408136 10.11607/ijp.5883

[16] T. Albrektsson , T. Jemt , J. Molne , P. Tengvall , and A. Wennerberg , “On Inflammation‐Immunological Balance Theory‐A Critical Apprehension of Disease Concepts Around Implants: Mucositis and Marginal Bone Loss May Represent Normal Conditions and Not Necessarily a State of Disease,” Clinical Implant Dentistry and Related Research 21 (2018): 183–189. 30592373 10.1111/cid.12711

[17] T. Albrektsson , C. Dahlin , D. Reinedahl , P. Tengvall , R. Trindade , and A. Wennerberg , “An Imbalance of the Immune System Instead of a Disease Behind Marginal Bone Loss Around Oral Implants: Position Paper,” International Journal of Oral & Maxillofacial Implants 35, no. 3 (2020): 495–502. 32406645 10.11607/jomi.8218

[18] R. Trindade , T. Albrektsson , P. Tengvall , and A. Wennerberg , “Foreign Body Reaction to Biomaterials: On Mechanisms for Buildup and Breakdown of Osseointegration,” Clinical Implant Dentistry and Related Research 18, no. 1 (2016): 192–203. 25257971 10.1111/cid.12274

[19] D. Hwang and H. L. Wang , “Medical Contraindications to Implant Therapy: Part I: Absolute Contraindications,” Implant Dentistry 15, no. 4 (2006): 353–360. 17172952 10.1097/01.id.0000247855.75691.03

[20] J. O. Esimekara , A. Perez , D. S. Courvoisier , and P. Scolozzi , “Dental Implants in Patients Suffering From Autoimmune Diseases: A Systematic Critical Review,” Journal of Stomatology, Oral and Maxillofacial Surgery 123, no. 5 (2022): e464–e473. 35033725 10.1016/j.jormas.2022.01.005

[21] A. Insua , A. Monje , H. L. Wang , and R. J. Miron , “Basis of Bone Metabolism Around Dental Implants During Osseointegration and Peri‐Implant Bone Loss,” Journal of Biomedical Materials Research. Part A 105, no. 7 (2017): 2075–2089. 28281321 10.1002/jbm.a.36060

[22] J. Wagner , J. H. Spille , J. Wiltfang , and H. Naujokat , “Systematic Review on Diabetes Mellitus and Dental Implants: An Update,” International Journal of Implant Dentistry 8, no. 1 (2022): 1. 34978649 10.1186/s40729-021-00399-8 PMC8724342

[23] B. R. Chrcanovic , T. Albrektsson , and A. Wennerberg , “Reasons for Failures of Oral Implants,” Journal of Oral Rehabilitation 41, no. 6 (2014): 443–476. 24612346 10.1111/joor.12157

[24] M. M. Bornstein , N. Cionca , and A. Mombelli , “Systemic Conditions and Treatments as Risks for Implant Therapy,” International Journal of Oral & Maxillofacial Implants 24, no. Suppl (2009): 12–27. 19885432

[25] G. Alsaadi , M. Quirynen , K. Michiles , W. Teughels , A. Komárek , and D. van Steenberghe , “Impact of Local and Systemic Factors on the Incidence of Failures up to Abutment Connection With Modified Surface Oral Implants,” Journal of Clinical Periodontology 35, no. 1 (2008): 51–57. 18034851 10.1111/j.1600-051X.2007.01165.x

[26] S. K. Chuang , L. J. Wei , C. W. Douglass , and T. B. Dodson , “Risk Factors for Dental Implant Failure: A Strategy for the Analysis of Clustered Failure‐Time Observations,” Journal of Dental Research 81, no. 8 (2002): 572–577. 12147750 10.1177/154405910208100814

[27] C. A. Bain and P. K. Moy , “The Association Between the Failure of Dental Implants and Cigarette Smoking,” International Journal of Oral & Maxillofacial Implants 8, no. 6 (1993): 609–615. 8181822

[28] B. R. Chrcanovic , T. Albrektsson , and A. Wennerberg , “Smoking and Dental Implants: A Systematic Review and Meta‐Analysis,” Journal of Dentistry 43, no. 5 (2015): 487–498. 25778741 10.1016/j.jdent.2015.03.003

[29] T. Jemt and U. Lekholm , “Implant Treatment in Edentulous Maxillae: A 5‐Year Follow‐Up Report on Patients With Different Degrees of Jaw Resorption,” International Journal of Oral & Maxillofacial Implants 10, no. 3 (1995): 303–311. 7615326

[30] R. D. Wåhlberg , V. F. Stenport , A. Wennerberg , and L. Hjalmarsson , “A Multicenter Study of Factors Related to Early Implant Failures‐Part 1: Implant Materials and Surgical Techniques,” Clinical Implant Dentistry and Related Research 27, no. 1 (2025): e70015. 39976277 10.1111/cid.70015 PMC11840881

[31] F. D'Ambrosio , A. Amato , A. Chiacchio , L. Sisalli , and F. Giordano , “Do Systemic Diseases and Medications Influence Dental Implant Osseointegration and Dental Implant Health? An Umbrella Review,” Dentistry Journal 11, no. 6 (2023): 146. 37366669 10.3390/dj11060146 PMC10296829

[32] M. Watanabe , L. Liu , and T. Ichikawa , “Are Allergy‐Induced Implant Failures Actually Hypersensitivity Reactions to Titanium? A Literature Review,” Dentistry Journal 11, no. 11 (2023): 263. 37999027 10.3390/dj11110263 PMC10670842

[33] U. Lekholm and G. Zarb , “Patient Selection and Preparation,” in Tissue‐Integrated Prostheses, ed. P.‐I. Branemark , G. Zarb , and T. Albrektsson (Quintessence, 1985).

[34] L. Amengual‐Peñafiel , L. A. Córdova , M. Constanza Jara‐Sepúlveda , M. Brañes‐Aroca , F. Marchesani‐Carrasco , and R. Cartes‐Velásquez , “Osteoimmunology Drives Dental Implant Osseointegration: A New Paradigm for Implant Dentistry,” Japanese Dental Science Review 57 (2021): 12–19. 33737990 10.1016/j.jdsr.2021.01.001 PMC7946347

[35] M. De Martinis , M. M. Sirufo , M. Suppa , and L. Ginaldi , “New Perspectives in Food Allergy,” International Journal of Molecular Sciences 21, no. 4 (2020): 1474. 32098244 10.3390/ijms21041474 PMC7073187

[36] Y. Ellenbogen , R. Jiménez‐Saiz , P. Spill , D. K. Chu , S. Waserman , and M. Jordana , “The Initiation of Th2 Immunity Towards Food Allergens,” International Journal of Molecular Sciences 19, no. 5 (2018): 1447. 29757238 10.3390/ijms19051447 PMC5983584

[37] J. Wang , Y. Zhou , H. Zhang , et al., “Pathogenesis of Allergic Diseases and Implications for Therapeutic Interventions,” Signal Transduction and Targeted Therapy 8, no. 1 (2023): 138. 36964157 10.1038/s41392-023-01344-4 PMC10039055

[38] W. Yu , D. M. H. Freeland , and K. C. Nadeau , “Food Allergy: Immune Mechanisms, Diagnosis and Immunotherapy,” Nature Reviews. Immunology 16, no. 12 (2016): 751–765. 10.1038/nri.2016.111 PMC5123910 27795547

[39] H. F. Rosenberg , K. D. Dyer , and P. S. Foster , “Eosinophils: Changing Perspectives in Health and Disease,” Nature Reviews. Immunology 13, no. 1 (2013): 9–22. 10.1038/nri3341 PMC4357492 23154224

[40] M. C. Berin , “Targeting Type 2 Immunity and the Future of Food Allergy Treatment,” Journal of Experimental Medicine 220, no. 4 (2023): e20221104, 10.1084/jem.20221104. 36880703 PMC9997511

[41] J. K. Nguyen , E. Austin , A. Huang , A. Mamalis , and J. Jagdeo , “The IL‐4/IL‐13 Axis in Skin Fibrosis and Scarring: Mechanistic Concepts and Therapeutic Targets,” Archives of Dermatological Research 312, no. 2 (2020): 81–92. 31493000 10.1007/s00403-019-01972-3 PMC7008089

[42] J. E. Allen , “IL‐4 and IL‐13: Regulators and Effectors of Wound Repair,” Annual Review of Immunology 41 (2023): 229–254. 10.1146/annurev-immunol-101921-041206 36737597

[43] S. Sayardoust , O. Omar , and P. Thomsen , “Gene Expression in Peri‐Implant Crevicular Fluid of Smokers and Nonsmokers. 1. The Early Phase of Osseointegration,” Clinical Implant Dentistry and Related Research 19, no. 4 (2017): 681–693. 28470893 10.1111/cid.12486

[44] I. Ghassib , Z. Chen , J. Zhu , and H. L. Wang , “Use of IL‐1 β, IL‐6, TNF‐α, and MMP‐8 Biomarkers to Distinguish Peri‐Implant Diseases: A Systematic Review and Meta‐Analysis,” Clinical Implant Dentistry and Related Research 21, no. 1 (2019): 190–207. 30508312 10.1111/cid.12694

[45] H. L. Yamaguchi , Y. Yamaguchi , and E. Peeva , “Role of Innate Immunity in Allergic Contact Dermatitis: An Update,” International Journal of Molecular Sciences 24, no. 16 (2023): 12975. 37629154 10.3390/ijms241612975 PMC10455292

[46] J. M. Zhang and J. An , “Cytokines, Inflammation, and Pain,” International Anesthesiology Clinics 45, no. 2 (2007): 27–37. 17426506 10.1097/AIA.0b013e318034194e PMC2785020

[47] T. Chaturvedi , “Allergy Related to Dental Implant and Its Clinical Significance,” Clinical, Cosmetic and Investigational Dentistry 5 (2013): 57–61. 23990733 10.2147/CCIDE.S35170 PMC3753052

[48] P. P. Poli , F. V. de Miranda , T. O. B. Polo , et al., “Titanium Allergy Caused by Dental Implants: A Systematic Literature Review and Case Report,” Materials (Basel) 14, no. 18 (2021): 5239. 34576463 10.3390/ma14185239 PMC8465040

[49] T. Harloff , W. Hönle , U. Holzwarth , R. Bader , P. Thomas , and A. Schuh , “Titanium Allergy or Not? “Impurity” of Titanium Implant Materials,” Health (NY) 2 (2010): 306–310.

[50] S. Bernard , M. Baeck , D. Tennstedt , V. Haufroid , and V. Dekeuleneer , “Chromate or Titanium Allergy—The Role of Impurities?,” Contact Dermatitis 68, no. 3 (2013): 191–192. 23421465 10.1111/cod.12024

[51] Tandvards och lakemedelsformansverket TLV , “Be Handling Med Implantat Blir Ersattningsberattigande Inom T and Position 6–6,” Historisk Utveckling av Statligt Tandvårdsstöd Med Konsekvensutredningar HUSK Website. 2014, https://husk.tlv.se/konsekvensutredning/2014_6/.

[52] T. Osterberg , G. E. Carlsson , and V. Sundh , “Trends and Prognoses of Dental Status in the Swedish Population: Analysis Based on Interviews in 1975 to 1997 by Statistics Sweden,” Acta Odontologica Scandinavica 58, no. 4 (2000): 177–182. 11045372 10.1080/000163500429181

[53] M. Chiapasco and M. Zaniboni , “Clinical Outcomes of GBR Procedures to Correct Peri‐Implant Dehiscences and Fenestrations: A Systematic Review,” Clinical Oral Implants Research 20, no. Suppl 4 (2009): 113–123. 19663958 10.1111/j.1600-0501.2009.01781.x

[54] T. Waller , M. Herzog , D. S. Thoma , J. Hüsler , C. H. F. Hämmerle , and R. E. Jung , “Long‐Term Clinical and Radiographic Results After Treatment or no Treatment of Small Buccal Bone Dehiscences at Posterior Dental Implants: A Randomized, Controlled Clinical Trial,” Clinical Oral Implants Research 31, no. 6 (2020): 517–525. 32011015 10.1111/clr.13588

[55] R. E. Jung , M. Herzog , K. Wolleb , C. F. Ramel , D. S. Thoma , and C. H. Hämmerle , “A Randomized Controlled Clinical Trial Comparing Small Buccal Dehiscence Defects Around Dental Implants Treated With Guided Bone Regeneration or Left for Spontaneous Healing,” Clinical Oral Implants Research 28, no. 3 (2017): 348–354. 26923088 10.1111/clr.12806

[56] C. H. F. Hämmerle and D. Tarnow , “The Etiology of Hard‐ and Soft‐Tissue Deficiencies at Dental Implants: A Narrative Review,” Journal of Periodontology 89, no. Suppl 1 (2018): S291–S303. 29926950 10.1002/JPER.16-0810

[57] T. Jemt , “Implant Survival in the Partially Edentulous Jaw 30 Years of Experience. Part III: A Retro‐Prospective Multivariate Regression Analysis on Overall Implant Failures in 2,915 Consecutively Treated Arches,” International Journal of Prosthodontics 32, no. 1 (2019): 36–44. 30677111 10.11607/ijp.5970

[58] G. Lin , S. Ye , F. Liu , and F. He , “A Retrospective Study of 30,959 Implants: Risk Factors Associated With Early and Late Implant Loss,” Journal of Clinical Periodontology 45, no. 6 (2018): 733–743. 29608788 10.1111/jcpe.12898

[59] I. Naert , G. Koutsikakis , J. Duyck , M. Quirynen , R. Jacobs , and D. van Steenberghe , “Biologic Outcome of Implant‐Supported Restorations in the Treatment of Partial Edentulism. Part I: A Longitudinal Clinical Evaluation,” Clinical Oral Implants Research 13, no. 4 (2002): 381–389. 12175375 10.1034/j.1600-0501.2002.130406.x

[60] A. A. Basson , J. Mann , M. Findler , and G. Chodick , “Correlates of Early Dental Implant Failure: A Retrospective Study,” International Journal of Oral & Maxillofacial Implants 38, no. 5 (2023): 897–906. 37847831 10.11607/jomi.10199

[61] R. Tabrizi , H. Behnia , S. Taherian , and N. Hesami , “What Are the Incidence and Factors Associated With Implant Fracture?,” Journal of Oral and Maxillofacial Surgery 75, no. 9 (2017): 1866–1872. 28623680 10.1016/j.joms.2017.05.014

[62] D. Buser , W. Martin , and U. C. Belser , “Optimizing Esthetics for Implant Restorations in the Anterior Maxilla: Anatomic and Surgical Considerations,” International Journal of Oral & Maxillofacial Implants 19, no. Suppl (2004): 43–61. 15635945

[63] A. Ramanauskaite and R. Sader , “Esthetic Complications in Implant Dentistry,” Periodontology 2000 88, no. 1 (2022): 73–85. 35103323 10.1111/prd.12412

[64] A. Kahn , D. Masri , T. Shalev , H. Meir , A. Sebaoun , and L. Chaushu , “Patients' Perception of Recovery After Dental Implant Placement,” Medicina (Kaunas, Lithuania) 57, no. 10 (2021): 1111. 34684148 10.3390/medicina57101111 PMC8538387

[65] Forsakringskassan , “Statistik om Tandvardsstod,” https://www.forsakringskassan.se/statistik‐och‐analys/statistik‐om‐tandvardsstod. 2025.

[66] L. Bohner , M. Hanisch , J. Kleinheinz , and S. Jung , “Dental Implants in Growing Patients: A Systematic Review,” British Journal of Oral & Maxillofacial Surgery 57, no. 5 (2019): 397–406. 31076220 10.1016/j.bjoms.2019.04.011

[67] F. Qiu , C. L. Liang , H. Liu , et al., “Impacts of Cigarette Smoking on Immune Responsiveness: Up and Down or Upside Down?,” Oncotarget 8, no. 1 (2017): 268–284. 27902485 10.18632/oncotarget.13613 PMC5352117

[68] L. Schwarz , V. Schiebel , M. Hof , C. Ulm , G. Watzek , and B. Pommer , “Risk Factors of Membrane Perforation and Postoperative Complications in Sinus Floor Elevation Surgery: Review of 407 Augmentation Procedures,” Journal of Oral and Maxillofacial Surgery 73, no. 7 (2015): 1275–1282. 25921824 10.1016/j.joms.2015.01.039

[69] A. Katranji , P. Fotek , and H. L. Wang , “Sinus Augmentation Complications: Etiology and Treatment,” Implant Dentistry 17, no. 3 (2008): 339–349. 18784534 10.1097/ID.0b013e3181815660

[70] E. M. Halawa , M. Fadel , M. W. Al‐Rabia , et al., “Antibiotic Action and Resistance: Updated Review of Mechanisms, Spread, Influencing Factors, and Alternative Approaches for Combating Resistance,” Frontiers in Pharmacology 14 (2023): 1305294. 38283841 10.3389/fphar.2023.1305294 PMC10820715

[71] A. Singh Gill , H. Morrissey , and A. Rahman , “A Systematic Review and Meta‐Analysis Evaluating Antibiotic Prophylaxis in Dental Implants and Extraction Procedures,” Medicina (Kaunas, Lithuania) 54, no. 6 (2018): 95. 30513764 10.3390/medicina54060095 PMC6306745

[72] T. Aghaloo , J. Pi‐Anfruns , A. Moshaverinia , D. Sim , T. Grogan , and D. Hadaya , “The Effects of Systemic Diseases and Medications on Implant Osseointegration: A Systematic Review,” International Journal of Oral & Maxillofacial Implants 34 (2019): s35–s49. 31116832 10.11607/jomi.19suppl.g3

